# The putative role of trigemino-vascular system in brain perfusion homeostasis and the significance of the migraine attack

**DOI:** 10.1007/s10072-022-06200-x

**Published:** 2022-07-08

**Authors:** Roberto De Simone, Mattia Sansone, Cinzia Russo, Angelo Miele, Antonio Stornaiuolo, Simone Braca

**Affiliations:** Department of Neuroscience, Reproductive Sciences and Odontostomatology, Headache Centre, University Federico II of Naples, Via Pansini, 5, 80122 Naples, Italy

**Keywords:** CGRP, PACAP, Idiopathic intracranial hypertension, Cerebral perfusion autoregulation, Migraine pathogenesis, Self-limiting venous collapse

## Abstract

Besides representing the place where a migraine attack generates, what is the physiological role of peptidergic control of arteriolar caliber within the trigemino-vascular system? Considering that the shared goal of most human CGRP-based neurosensory systems is the protection from an acute threat, especially if hypoxic, what is the end meaning of a migraine attack? In this paper, we have reviewed available evidence on the possible role of the trigemino-vascular system in maintaining cerebral perfusion pressure homeostasis, despite the large physiological fluctuations in intracranial pressure occurring in daily life activities. In this perspective, the migraine attack is presented as the response to a cerebral hypoxic threat consequent to a deranged intracranial pressure control aimed at generating a temporary withdrawal from the environment with limitation of physical activity, a condition required to promote the restoration of cerebral fluids dynamic balance.

## Introduction

Research over the last few decades has identified and largely understood the crucial role of the trigemino-vascular system (TVS) and the calcitonin gene-related peptide (CGRP) in generating migraine pain [1]. However, some questions have not yet been answered satisfactorily.

1. The first question refers to the actual physiological significance of the precise peptidergic control of the arteriolar caliber within the TVS. Besides representing the place where a migraine attack generates, what is the function of the TVS between attacks or in non-migraine sufferers? What are the physiological needs that require its presence, in addition to the rich biochemical and neuromediated orthosympathetic and parasympathetic regulation of the cerebral vascular districts?
2. What is the pathophysiologic significance of the migraine attack? Nociceptive activation responds to a threat to the integrity of the organism or to its biological homeostasis, resulting from pathological dysfunctions or from the organism’s interaction with the changing environment [2]. Today we look at pain not only as a conscious sensation, useful for localizing the stimulus and identifying its nature, but also as a starting point for triggering a much more complex response that implies a cognitive and affective processing of incoming inputs and the activation of specific behaviors aimed at avoiding the threat or generating the best conditions to recover from the perturbed biological homeostasis [3, 4]. This justifies complexity and richness of the cortical and subcortical areas involved in the central processing of pain included in the so-called pain matrix, in turn integrated into the “vegetative brain” which oversees these functions, represented in the limbic system [5]. In keep with this, the withdrawal from any activity and the avoidance of sensorial stimuli and movement that characterize the migraine attack very closely recall the mammalian “sickness behavior,” typically evoked by *unescapable* visceral pain and aimed at promoting the temporary disengagement of the organism from the external environment [4, 6]. If we look at migraine pain as the trigger of a complex innate defensive response, such as sickness behavior, we need to ask ourselves what is the nature of the threat to which the migraine attack represents the more or less appropriate response.

In this paper, we will try, speculatively, to answer the above two questions.

## CGRP-based neurosensory systems

The TVS is not the only CGRP-based neurosensory system. There are several other vegetative sensory systems that trigger CGRP-dependent vasodilation in response to potential harm. The visceral neurosensorial afferent systems operate at enteric tube level and particularly in the gastric mucosa [7, 8]. Afferent sensitive spinal fibers, expressing the capsaicin transient receptor potential vanilloid-1 (TRPV-1), reach the gastric mucosa. Besides capsaicin, they are sensitive to hydrochloric acid, ethanol, bile salts, aspirin, indomethacin, and other potentially harmful factors. These fibers send extensions to the submucosal vascular network. When activated, they trigger CGRP and substance P (SP) release with subsequent increase in blood flow and vessels permeability of the mucosa, of defensive significance [7, 8]. The reflex vasodilation is responsible for a greater resistance of the gastric mucosa to a series of pathogens in subjects exposed for cultural reasons to a diet rich in capsaicin, probably due to a more frequent activation of this defensive reflex. The incidence of ulcer disease in the population of Singapore inversely correlates with the amount of chili intake [9]. A similar phenomenon also occurs in the skin, where a CGRP-dependent vasodilation can occur as a result of various nociceptive stimuli and contributes to the wound healing [10], as well as at the cardiac level, where CGRP shows direct anti-ischemic actions and is released as an emergency reaction to an acute ischemia [11]. The relevance of CGRP as a response to ischemic conditions is also suggested by the known neoangiogenic properties of this peptide [12].

### Beyond the CGRP: the pituitary adenylato cyclase activating polipeptide

The vasomotor peptides released by TVS and implicated in the pathogenesis of migraine include the pituitary adenylato cyclase activating polypeptide (PACAP) [13]. Exogenous administration of PACAP induces a typical migraine attack only in migraine patients [14], as observed for CGRP [15]. The involvement of PACAP in migraine mechanisms is not limited to vasodilation, but might include dural mast cell degranulation [16]. Moreover, PACAP promotes cortisol and cathecolamine secretion in response to stress [17] and modulates specific cellular functions involved in cellular resistance to anoxia and in inhibition of apoptosis, ultimately providing neuroprotection from hypoxia [18]. Anti-ischemic properties of PACAP can be observed not only in the nervous system but also in several other organs included the liver, small intestine, and kidney suggesting a general anti-ischemic protective role of this peptide [18].

Thus, peptidergic neurosensory system activation represents at many levels the response to a potentially harmful threat. Their direct powerful vasodilatory action and indirect hypoxia protective effects suggest that reduced tissue perfusion may be a shared trigger for their activation.

## Is ischemia involved in migraine?

Migraine, especially with aura, is associated with increased cerebrovascular risk [19–21], occasionally with ischemic events [22] and with increased hypertensive risk [23]. Gliotic-like white matter lesions (WML) characterize the migraine brain in the youth and adult age groups with an estimated prevalence of 70% in vascular risk factor-free migraine patients aged < 50 years [24, 25]. There is growing evidence that deep WML express altered cerebral hemodynamic suggesting their hypoxic origin [24–26]. The hypothesis that a brief episode of cerebral hypoxia occurs in any migraine attack was advanced by Amery as early as 1982 [27]. Prevalence of migraine, particularly with aura, is greatly increased in communities living at very high altitude, thus in conditions of significant hypobaric hypoxia. In an epidemiological study on a male community living at 4300 m above sea level, migraine was present in 32% of subjects and aura was reported in the majority of the cases [28]. It has been shown that exposure of migraine subjects to normobaric hypoxia can trigger both the migraine attacks and the manifestations of the aura [29]. These findings have been replicated in a recent study [30] in which aura occurred for the first time under experimental hypoxic conditions also in two migraine without aura volunteers. These studies agree in considering hypoxia a powerful trigger of migraine attack with or without aura.

## The striking similarities between migraine and Idiopathic intracranial hypertension headache

Migraine has numerous similarities with idiopathic intracranial hypertension headache with (IIH) or without papilledema (IIHWOP). It is known that the two conditions are completely indistinguishable on clinical basis, so that an IIHWOP is found between 10 and 86% of chronic migraine (CM) series [31–34]. They also share main risk factors (obesity, female sex, sleep disorders) [35], some treatments such as topiramate [36] but also migraine-specific treatments as MAb anti-CGRP Receptor [37], suggesting the causative involvement of CGRP also in IIH/IIHWOP-related headache. Finally, both conditions show a high prevalence of significant dural sinus stenosis [38, 39], considered a neuro-radiological marker of IIH/IIHWOP [40]. These similarities indicate a close pathogenetic link between CM and IIHWOP and that a derangement of intracranial pressure (ICp) control associated with dural sinus stenosis is probably a shared mechanism between the two. There is evidence that sinus stenosis-associated increased ICp is a very common condition in the general population, found in up to 11.1% of individuals without persistent headache or other symptoms or signs of IIH/IIHWOP [41]. Their prevalence raises to 44.8% in unselected chronic headache series [38] and up to 86% in chronic migraine cases with prospectively assessed refractoriness [33]. On this basis, it has been proposed [35] that an unacknowledged IIHWOP associated with sinus stenosis can occur almost asymptomatically in many individuals but that in subjects with a predisposition to migraine, it represents a powerful and modifiable [33, 34] risk factor for the progression and refractoriness of migraine [42]. IIHWOP was recently mentioned in a reference paper on chronic migraine risk factors [43].

IIH/IIHWOP is associated with profound cerebral fluid dynamics rearrangements such as redistribution of venous flow towards extracranial, epidural, and vertebral veins [44]; increase in resistivity [45, 46] and pulsatility indices [46]; arterial hypoperfusion [47]; and reduced cerebrovascular response to hypoxia [25] which resolve after treatment [19, 46]. Could the impact of IIH on cerebral fluid dynamics imply a perfusion risk? Ideally, relocating the pathogenesis of migraine in the context of IIH/IIHWOP could allow us to attempt an answer to our initial questions. However, to illustrate the putative role played by TVS in the control of cerebral perfusion and to propose the possible finalistic significance of the migraine attack, it is necessary to summarize some mechanisms involved in cerebral perfusion and CSF turnover dynamics which occur on the venous side of the cerebral circulation [48].

## Cerebral perfusion and CSF turnover integrated dynamics

The cerebral perfusion dynamics and those that govern CSF turnover can mutually influence each other and require to be described in an integrated way [48].

### Routes of CSF excretion

The dural sinus is not the only CSF exit route. The recently identified glymphatic system [49] and the CSF reabsorption occurring at cranial and spinal nerves levels significantly contribute to the overall interstitial fluid/CSF discharge [50]. Although quantitatively the reabsorption of CSF through the dural sinuses is no longer considered the more significant, the alternative CSF exit routes are probably easily saturable [51] and the glymphatic system may become inefficient in subjects with increased intracranial pressure [52]. On the contrary, the CSF discharge rate through arachnoid villi and granulations *linearly* correlates with the CSF/dural sinus transmural pressure gradient, up to values ​that exceed the CSF production rate by several times [53]. Therefore, CSF discharge at dural sinus level may quickly counteract the physiologic intracranial pressure peaks associated with posture and movement, a mechanism that prevents the overflow of accessory (glymphatic / spinal) CSF discharge routes, with also a crucial role in CSF volume and pressure stabilization [54].

### Fluid dynamic constrains of cerebral perfusion physiology

The physiological balance of cerebral perfusion implies that the pressures of the intracranial fluids, while physiologically fluctuating, respect a rigid pressure hierarchy [55]. The arteriolar pressure entering the cerebral perfusion circuit (Ap) is always higher than the cortical veins pressure (CVp), this difference reflecting the cerebral perfusion pressure (Pp) on which the cerebral flow depends. In turn, the CVp is dynamically maintained always just above the intracranial pressure to prevent the large physiological fluctuations of the latter (due to postural changes or the Valsalva effect) from compressing the thin cortical veins, hindering the flow [56–58]. At the same time, the ICp always remains higher than the pressure in the dural sinuses (DSp), a condition required for the CSF discharge to remain adequate and balanced with its own production [57–59]. This implies that a pressure drop must occur between the cortical vein and the dural sinus despite their physical continuity so that intracranial pressure is always maintained between the cortical veins pressure and the dural sinuses pressure. Actually, an abrupt venous pressure drop has been directly observed at the confluence between the bridge vein (BV) — i.e., the terminal segment of cortical vein — and the dural sinus using a catheter passed in and out the venous confluence, in dog models [57, 60].

### The pivotal role of bridge vein

**Fig. 1:**
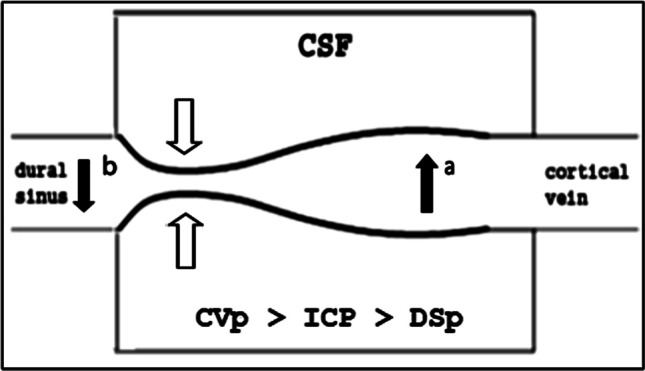
The twofold effect of the bridge vein collapse under raised ICp (white arrows): increase of cortical vein pressure upstream (black arrow **a**); decrease of the dural sinus pressure downstream (black arrow **b**)

The rigorous respect of these fluid-dynamic constraints (Ap > CVp > ICp > DSp) despite the large physiological fluctuations of the ICp is ensured by a fascinating mechanism that occurs at BV level. The BV behaves like a “Starling resistor” (SR), a fluid-dynamic construct that describes the flow in collapsible tubes immersed in a fluid at variable pressure [61]. Due to the SR properties of BV, its caliber reduces when the ICp increases, in proportion to the raised transmural pressure. The effect of BV narrowing is twofold: on the one hand, the pressure of the upstream cortical vein is proportionally increased (Fig. 1), in real time and with high linear correlation with ICp (*r* = 0.98) [57, 58] beyond the value of the ICp so as to prevent its compression (that would reduce the blood flow). On the other hand, a sudden drop in dural sinus pressure is generated (“waterfall effect” [62]) (Fig. 1): once passed the BV, the blood pressure does not reflect anymore the cardiac *vis a tergo* [57, 60] reflecting instead the right atrial pressure downstream, lower and more stable. The waterfall effect guarantees the maintenance of the correct CSF/dural sinus pressure gradient required for CSF absorption. The dual effect of the Starling resistor mechanism of the bridging veins therefore plays a crucial role in the balance between cerebral perfusion and CSF turnover dynamic, ensuring the respect of cerebral fluid pressure hierarchies and representing a point of mutual interaction and balance between blood and CSF circulations.

### Rigidity of dural sinus and IIH/IIHWOP pathogenesis

**Fig. 2:**
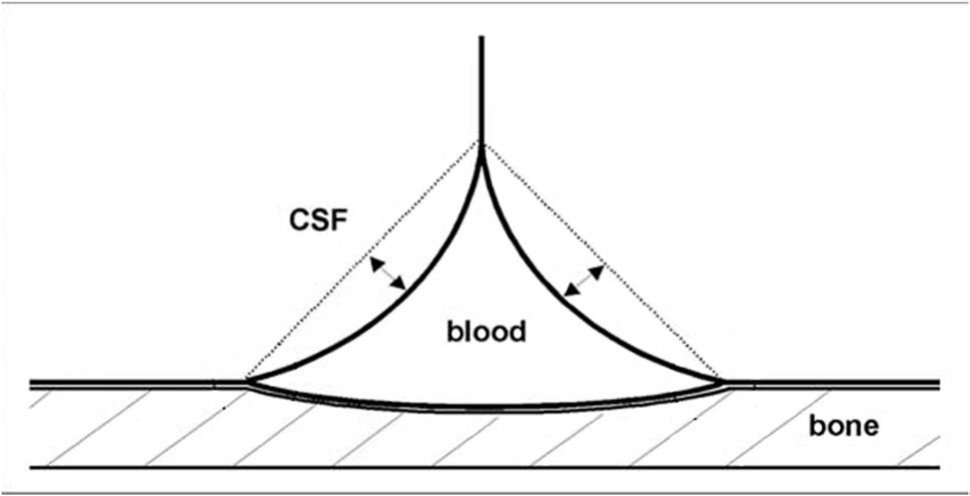
Collapsibility of dural sinus is limited by their prismatic shape, with a side attached to the bone

**Fig. 3:**
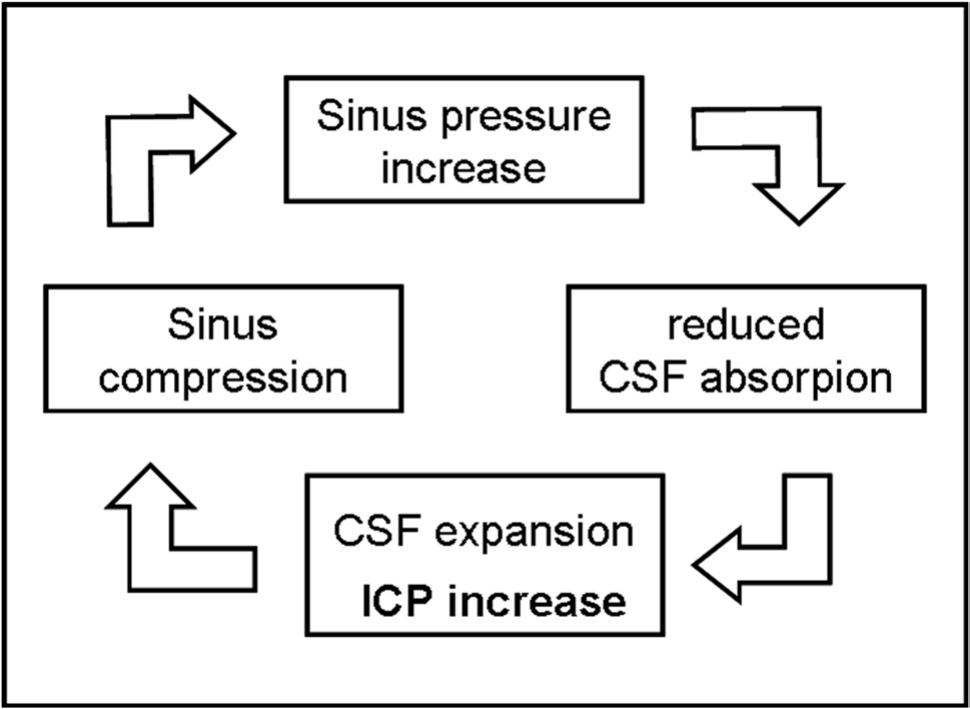
The Self-limiting Venous Collapse feedback loop

These mechanisms presuppose the substantial rigidity of the dural sinuses, which is essential for the physiological fluctuations of the ICp (under Valsalva maneuver up to 470 mmH_2_O [63]) not to be transmitted to the sinus, compromising the waterfall effect and consequently the CSF reabsorption rate. The rigidity of the dural sinuses is entrusted to their triangular section with one side firmly anchored to the bone (Fig. 2) but also to a documented greater mechanical resistance of the dura mater that lines the dural sinuses compared to other areas, due to a greater density of collagen 1 [64]. We have proposed that a reduced rigidity of dural sinus is causatively involved in IIH/IIHWOP mechanisms due to a “self-limiting venous collapse feedback loop” between the CSF pressure, which compresses the excessively compliant sinus, and the consequent rise in sinus pressure which, by reducing the CSF absorption, leads to an increase in the CSF volume and pressure with further sinus compression [65] (Fig. 3). The pathologic coupling of the intracranial pressure with the dural sinuses pressure has been directly observed in a recent human study on IIH patients [66]. The loop stops when the maximum venous compression is reached. Then, further CSF production can restore the transmural CSF/dural blood pressure required to balance CSF excretion with its production rate. A new equilibrium is thus achieved, at the price of higher and more unstable intracranial and dural sinus pressures [65].

### The need for an arteriolar pressure compensation

The perfusion pressure (Pp) is the difference between the inlet and outlet pressure of the cerebral perfusion circuit but it is calculated as the differential between the mean arterial pressure and the intracranial pressure due to the strict coupling of cortical vein pressure and ICp at bridge vein level, with the latter easier to monitor in clinical settings. The perfusion pressure is approximately 50–70 mmHg [67]. The cerebral blood flow remains proportional to Pp, which therefore must remain unchanged despite the continuous physiological fluctuations of absolute values ​of the two pressures that determine it. The raise of the cortical vein pressure induced by the intracranial pressure increase prevents the thin cortical vein wall from being compressed. However, this dynamic would reduce the cerebral perfusion pressure, and thus the blood flow, if it were not promptly compensated by a corresponding and almost simultaneous increase in arteriolar caliber, which keeps the perfusion pressure unchanged. Convergent evidence indicates that arteriolar dilation occurs when intracranial pressure increases [56, 68–72]. In an important work on the baboon in which the pressures of the intracranial vascular districts were measured invasively, a sharp drop in arteriolar resistance was documented in response to the experimentally induced increase in intracranial pressure [56]. A significant dilation of the cortico-pial arterioles resulting from the experimental increase in ICp was directly observed in an animal study using cranial fenestration and videoangiometry techniques [68]. At ICp of 50 mmHg, the arteriolar caliber increased by 33 ± 3% in larger arterioles and by 42 ± 5.6% in smaller arterioles. Transcranial Doppler (TCD) studies performed during continuous intracranial pressure monitoring in subjects with suspected normal pressure hydrocephalus [69] or in head injured patients [70] demonstrated that the blood velocity of middle cerebral artery (MCA) continuously fluctuates along spontaneous B-waves strictly in phase with the ICp oscillations indicating that arterioles behind the MCA dilates along the B-wave [71]. Similar TCD periodic fluctuations of MCA blood velocity have been documented in healthy subjects [72].

Indeed, a strict coupling of the intracranial pressure with the arteriolar caliber do exists and guaranties the constancy of the cerebral perfusion pressure despite the continuous physiological ICp fluctuations occurring in daily life. Is TVS involved in such a critical function?

## The putative role of TVS in the dynamic coupling of the outlet pressure with the inlet pressure of the brain perfusional circuit

The TVS is distributed to both arterial and venous intracranial vessels primarily through the peripheral endings of the first branch of the Gasser ganglion. Although its pathophysiological role in the genesis of the migraine attack has been studied mainly at the arterial level, we know since the pioneering studies by Ray and Wolff [73] that the site with the greatest sensitivity to pain is the dura that covers the venous sinuses. A recent postmortem human study on the superior sagittal sinus has shown that these districts are richly innervated by TVS fibers and that the main peptide released by these terminals is CGRP. Ruffini-like stretching receptors were also found especially at the level of the confluences of the BV with dural sinus, probably meaning that the external pressure-dependent dural stretching of the complex bridge vein-dural sinus may physiologically act as a sensor of the intracranial pressure and, indirectly, of the cortical vein pressure [74, 75]. On this basis, TVS could be involved in the control of cerebral perfusion with the crucial role of maintaining dynamically coupled the fluctuating venous outlet pressure with the inlet arterial pressure, thus, to maintain a constant perfusion pressure despite the sudden increases in ICp due to postural changes or Valsalva effect. In line with this, the powerful vasodilating property and the short half-life of CGRP [76] and PACAP [77] seem adequate to generate a prompt fine-tuning of the arteriolar caliber. The mechanism could be biohumoral and take place through the release into the blood of vasodilating peptides. However, TVS could have among its physiological functions also the ICp monitoring and the neurally mediated compensatory modulation of the arteriolar tone. Under physiological conditions, this mechanism would not involve activation of nociception while it would ensure the maintenance of an adequate perfusion pressure in all circumstances required by the dynamic interaction between the organism and the environment. This function might be regarded as an ancient homeostatic process of brain perfusion probably contributing to the achievement of upright posture in primates.

## The putative significance of the migraine attack

Following this perspective, in subjects with derangement of intracranial pressure control associated with collapsible dural sinus, a headache attack could signal the exceeding of a critical ICp threshold and expresses the risk of an imminent impairment of cerebral perfusion due to the malfunction or to the exceeding of the TVS compensatory abilities. The painful attack would therefore respond to a hypoxic threat and would have the purpose of imposing disengagement from the environment and the cessation of all physical activity (which is associated with fluctuations in the intracranial pressure) for a time sufficient to allow the stabilization of intracranial pressure and cerebral perfusion. It is also conceivable that a top-down modulation of the TVS activation threshold under the control of limbic supraspinal structures projecting to trigeminal nucleus might promote the triggering of the attack even in the presence of ordinary trigemino-vascular sensory traffic [78]. Such a mechanism could allow the activation of a migraine attack by the limbic systems in response to other kind of threats to body homeostasis which nonetheless require a similar temporary withdrawal from the environment and daily activities. However, even in these cases, the mechanical stress of the vascular walls generated by the interactions between ICp changes and venous discharge into dural sinus at the BV level would probably represent the main neurosensory input from which the cascade of events leading to the migraine attack moves.

## Is migraine a primary or a secondary condition?

Intracranial pressure is unstable and tends to be higher in many individuals who share disturbances in intracranial venous discharge [41]. However, according to a large multicentric study, IIH may present without headache in 15.8% of cases and with non-classifiable headache in 7.2%, with tension-type or probable tension-type headache in 25.1% and with migraine or probable migraine (episodic or chronic) in 67.6% of the headache cases [79]. This “continuum” suggests that a primary predisposition to migraine pain is required for the increased intracranial pressure to produce the typical migraine manifestations, and also modulates frequency and intensity of attacks. Headache-free IIH has been observed in subjects with no personal or family history of migraine or in the course of a migraine protective factor such as pregnancy [80]. The nature of this predisposition to migraine pain is probably multifactorial and polygenic and it can vary between different individuals and within the same individual over time. It could be linked to the multiple peculiarities of the “migraine brain” [81], to a lower allodynic threshold [82], might be modulated by estrogen fluctuations in female sex, and can explain the observation that the CGRP or PACAP induce a migraine attack only in migraine patients [14, 15]. In this perspective, migraine remains a “primary” disease that afflicts a minority of the individuals carrying a sinus stenosis-associated deranged intracranial pressure control, which also shares the ability to trigger a migraine attack at relatively low trigemino-vascular activation threshold.

## Conclusions

The TVS is the most likely candidate for the role of main coordinator of the inlet and outlet pressures coupling of cerebral perfusion circuit. This function is crucial for the maintenance of the constancy of perfusion in response to the large fluctuations of the intracranial pressure that occur physiologically with postural changes or physical activities involving the Valsalva effect and that reflect on cortical vein pressure due to the SR properties of BV. In physiological conditions of perfusion, the TVS would not generate paroxysmal activations or abnormal release of CGRP, but would nevertheless always be active as evidenced by the detectable amount of alpha-CGRP in peripheral blood (even in non-migraineurs). In subjects with collapsing dural sinus, the amplitude of the ICp fluctuations can be very large. Upon exceeding an individual “alarm” threshold indicating an impending threat to the cerebral perfusion, a massive release of CGRP aimed to restoring an adequate Pp occurs, ultimately leading to the migraine attack in subjects also sharing a primary predisposition to migraine pain.

## References

[1] Lyengar S, Johnson KW, Ossipov MH, Aurora SK (2019). CGRP and the trigeminal system in migraine. Headache.

[2] Craig AD (2003). A new view of pain as a homeostatic emotion. Trends Neurosci.

[3] Craig AD (2002). (2002) How do you feel? Interoception: the sense of the physiological condition of the body. Nat Rev Neurosci.

[4] Bonavita V, De Simone R (2011). Pain as an evolutionary necessity. Neurol Sci.

[5] Thompson JM, Neugebauer V (2019). Cortico-limbic pain mechanisms. Neurosci Lett.

[6] Montagna P, Pierangeli G, Cortelli P (2010). The primary headaches as a reflection of genetic darwinian adaptive behavioral responses. Headache.

[7] Holzer P, Pabst MA (1999). Visceral afferent neurons: role in gastric mucosal protection. News Physiol Sci.

[8] Holzer P (1998). Neural emergency system in the stomach. Gastroenterology.

[9] Kang JY, Yeoh KG, Chia HP (1995). Chili–protective factor against peptic ulcer?. Dig Dis Sci.

[10] Wurthmann S, Nägel S, Hadaschik E (2020). Impaired wound healing in a migraine patient as a possible side effect of calcitonin gene-related peptide receptor antibody treatment: a case report. Cephalalgia.

[11] Kee Z, Kodji X, Brain SD (2018). The role of calcitonin gene related peptide (CGRP) in neurogenic vasodilation and its cardioprotective effects. Front Physiol.

[12] Zheng S, Li W, Xu M (2010). Calcitonin gene-related peptide promotes angiogenesis via AMP-activated protein kinase. Am J Physiol Cell Physiol.

[13] Edvinsson L, Tajti J, Szalárdy L, Vécsei L (2018). PACAP and its role in primary headaches. J Headache Pain.

[14] Schytz HW, Birk S, Wienecke T (2009). PACAP38 induces migraine-like attacks in patients with migraine without aura. Brain.

[15] Hansen JM, Hauge AW, Olesen J, Ashina M (2010). Calcitonin gene-related peptide triggers migraine-like attacks in patients with migraine with aura. Cephalalgia.

[16] Baun M, Pedersen MHF, Olesen J, Jansen-Olesen I (2012). Dural mast cell degranulation is a putative mechanism for headache induced by PACAP-38. Cephalalgia.

[17] Stroth N, Holighaus Y, Ait-Ali D, Eiden LE (2011). PACAP: a master regulator of neuroendocrine stress circuits and the cellular stress response. Ann N Y Acad Sci.

[18] Reglodi D, Vaczy A, Rubio-Beltran E, MaassenVanDenBrink A (2018). Protective effects of PACAP in ischemia. J Headache Pain.

[19] Mahmoud AN, Mentias A, Elgendy AY (2018). Migraine and the risk of cardiovascular and cerebrovascular events: a meta-analysis of 16 cohort studies including 1 152 407 subjects. BMJ Open.

[20] Øie LR, Øie LR, Kurth T (2020). Migraine and risk of stroke. J Neurol Neurosurg Psychiatry.

[21] Kurth T, Rist PM, Ridker PM (2020). Association of migraine with aura and other risk factors with incident cardiovascular disease in women. JAMA.

[22] Laurell K, Artto V, Bendtsen L (2011). Migrainous infarction: a Nordic multicenter study. Eur J Neurol.

[23] Rist PM, Winter AC, Buring JE (2018). Migraine and the risk of incident hypertension among women. Cephalalgia.

[24] Cheng CY, Cheng HM, Chen SP (2018). White matter hyperintensities in migraine: clinical significance and central pulsatile hemodynamic correlates. Cephalalgia.

[25] Lee MJ, Park BY, Cho S (2019). Cerebrovascular reactivity as a determinant of deep white matter hyperintensities in migraine. Neurology.

[26] Zhang Q, Datta R, Detre JA, Cucchiara B (2017). White matter lesion burden in migraine with aura may be associated with reduced cerebral blood flow. Cephalalgia.

[27] Amery WK (1982). Brain hypoxia: the turning-point in the genesis of the migraine attack?. Cephalalgia.

[28] Arregui A, León-Velarde F, Cabrera J (1994). Migraine, polycythemia and chronic mountain sickness. Cephalalgia.

[29] Arngrim N, Schytz HW, Britze J (2016). Migraine induced by hypoxia: an MRI spectroscopy and angiography study. Brain.

[30] Frank F, Faulhaber M, Messlinger K (2020). Migraine and aura triggered by normobaric hypoxia. Cephalalgia.

[31] Mathew NT, Ravishankar K, Sanin LC (1996). Coexistence of migraine and idiopathic intracranial hypertension without papilledema. Neurology.

[32] Vieira DSS, Masruha MR, Gonçalves AL (2008). Idiopathic intracranial hypertension with and without papilloedema in a consecutive series of patients with chronic migraine. Cephalalgia.

[33] De Simone R, Ranieri A, Montella S (2014). Intracranial pressure in unresponsive chronic migraine. J Neurol.

[34] Favoni V, Pierangeli G, Toni F (2018). Idiopathic intracranial hypertension without papilledema (IIHWOP) in chronic refractory headache. Front Neurol.

[35] De Simone R, Ranieri A, Fiorillo C (2010). Is idiopathic intracranial hypertension without papilledema a risk factor for migraine progression?. Neurol Sci.

[36] Çelebisoy N, Gökçay F, Şirin H, Akyürekli Ö (2007). Treatment of idiopathic intracranial hypertension: topiramate vs acetazolamide, an open-label study. Acta Neurol Scand.

[37] Yiangou A, Mitchell JL, Vijay V (2020). Calcitonin gene related peptide monoclonal antibody treats headache in patients with active idiopathic intracranial hypertension. J Headache Pain.

[38] Bono F, Salvino D, Tallarico T (2010). Abnormal pressure waves in headache sufferers with bilateral transverse sinus stenosis. Cephalalgia.

[39] Farb RI, Vanek I, Scott JN (2003). Idiopathic intracranial hypertension: the prevalence and morphology of sinovenous stenosis. Neurology.

[40] Morris PP, Black DF, Port J, Campeau N (2017). Transverse sinus stenosis is the most sensitive MR imaging correlate of idiopathic intracranial hypertension. AJNR Am J Neuroradiol.

[41] Bono F, Cristiano D, Mastrandrea C (2010). The upper limit of normal CSF opening pressure is related to bilateral transverse sinus stenosis in headache sufferers. Cephalalgia.

[42] De Simone R, Ranieri A, Cardillo G, Bonavita V (2011). High prevalence of bilateral transverse sinus stenosis-associated IIHWOP in unresponsive chronic headache sufferers: pathogenetic implications in primary headache progression. Cephalalgia.

[43] Buse DC, Greisman JD, Baigi K, Lipton RB (2019). Migraine progression: a systematic review. Headache.

[44] Alperin N, Lee SH, Mazda M (2005). Evidence for the importance of extracranial venous flow in patients with idiopathic intracranial hypertension (IIH). Acta Neurochir Suppl.

[45] Mohammaden MH, Husain MR, Brunozzi D (2020). Role of resistivity index analysis in the prediction of hemodynamically significant venous sinus stenosis in patient with idiopathic intracranial hypertension. Neurosurgery.

[46] Juhász J, Lindner T, Jansen O (2018). Changes in intracranial venous hemodynamics in a patient with idiopathic intracranial hypertension after lumbar puncture precedes therapeutic success. J Magn Reson Imaging.

[47] Ding J, Guan J, Ji X, Meng R (2020). Cerebral venous sinus stenosis may cause intracranial arterial hypoperfusion. Clin Neuroradiol.

[48] De Simone R, Ranieri A, Bonavita V (2017). Starling resistors, autoregulation of cerebral perfusion and the pathogenesis of idiopathic intracranial hypertension. Panminerva Med.

[49] Hablitz LM, Nedergaard M (2021). The glymphatic system. Curr Biol.

[50] Proulx ST (2021). Cerebrospinal fluid outflow: a review of the historical and contemporary evidence for arachnoid villi, perineural routes, and dural lymphatics. Cell Mol Life Sci.

[51] Lenck S, Radovanovic I, Nicholson P, Hodaie M, Krings T, Mendes-Pereira V (2018). Idiopathic intracranial hypertension: the veno glymphatic connections. Neurology.

[52] Eide PK, Pripp AH, Ringstad G, Valnes LM (2021) Impaired glymphatic function in idiopathic intracranial hypertension. Brain Commun 3(2):fcab043 10.1093/braincomms/fcab043 PMC8253298 34235434

[53] Albeck MJ, Borgesen SE, Gjerris F (1991). Intracranial pressure and cerebrospinal fluid outflow conductance in healthy subjects. J Neurosurg.

[54] Leishangthem L, SirDeshpande P, Dua D, Satti SR (2019). Dural venous sinus stenting for idiopathic intracranial hypertension: an updated review. J Neuroradiol.

[55] Barami K, Sood S (2016). The cerebral venous system and the postural regulation of intracranial pressure: implications in the management of patients with cerebrospinal fluid diversion. Childs Nerv Syst.

[56] Johnston IH, Rowan JO (1974). Raised intracranial pressure and cerebral blood flow. 3. Venous outflow tract pressures and vascular resistances in experimental intracranial hypertension. J Neurol Neurosurg Psychiatry.

[57] Nakagawa Y, Tsuru M, Yada K (1974). Site and mechanism for compression of the venous system during experimental intracranial hypertension. J Neurosurg.

[58] Yada K, Nakagawa Y, Tsuru M (1973). Circulatory disturbance of the venous system during experimental intracranial hypertension. J Neurosurg.

[59] Shulman K, Yarnell P, Ransohoff J (1964). Dural sinus pressure: in normal and hydrocephalic dogs. Arch Neurol.

[60] Luce JM, Huseby JS, Kirk W, Butler J (1982). A Starling resistor regulates cerebral venous outflow in dogs. J Appl Physiol.

[61] Morgan P, Parker KH (1989). A mathematical model of flow through a collapsible tube-I. Model and steady flow results. J Biomech.

[62] Permutt S, Riley RL (1963). Hemodynamics of collapsible vessels with tone: the vascular waterfall. J Appl Physiol.

[63] Neville L, Egan RA (2005). Frequency and amplitude of elevation of cerebrospinal fluid resting pressure by the Valsalva maneuver. Can J Ophthalmol.

[64] Walsh DR, Ross AM, Malijauskaite S (2018). Regional mechanical and biochemical properties of the porcine cortical meninges. Acta Biomater.

[65] De Simone R, Ranieri A, Sansone M (2019). Dural sinus collapsibility, idiopathic intracranial hypertension, and the pathogenesis of chronic migraine. Neurol Sci.

[66] Lalou AD, Czosnyka M, Czosnyka ZH (2020). Coupling of CSF and sagittal sinus pressure in adult patients with pseudotumour cerebri. Acta Neurochir (Wien).

[67] Tamm AS, McCourt R, Gould B (2016). Cerebral perfusion pressure is maintained in acute intracerebral hemorrhage: a CT perfusion study. Am J Neuroradiol.

[68] Auer LM, Ishivama N, Pucher R (1987). Cerebrovascular response to intracranial hypertension. Acta Neurochir (Wien).

[69] Droste DW, Krauss JK (1993). Simultaneous recording of cerebrospinal fluid pressure and middle cerebral artery blood flow velocity in patients with suspected symptomatic normal pressure hydrocephalus. J Neurol Neurosurg Psychiatry.

[70] Newell DW, Aaslid R, Stooss R, Reulen HJ (1992). The relationship of blood flow velocity fluctuations to intracranial pressure B waves. J Neurosurg.

[71] Lundberg N (1960). Continuous recording and control of ventricular fluid pressure in neurosurgical practice. Acta Psychiatr Scand Suppl.

[72] Droste DW, Krauss JK, Berger W (1994). Rhythmic oscillations with a wavelength of 0.5–2 min in transcranial Doppler recordings. Acta Neurol Scand.

[73] Ray BS, Wolff HG (1940). Experimental studies on headache pain-sensitive structures of the head and their significance in headache. Arch Surg.

[74] Andres KH, von Düring M, Muszynski K, Schmidt RF (1987). Nerve fibres and their terminals of the dura mater encephali of the rat. Anat Embryol (Berl).

[75] Sampaolo S, Liguori G, Vittoria A (2017). First study on the peptidergic innervation of the brain superior sagittal sinus in humans. Neuropeptides.

[76] Kraenzlin ME, Ch’ng JLC, Mulderry PK,  (1985). Infusion of a novel peptide, calcitonin gene-related peptide (CGRP) in man. Pharmacokinetics and effects on gastric acid secretion and on gastrointestinal hormones. Regul Pept.

[77] Bourgault S, Vaudry D, Botia B (2008). Novel stable PACAP analogs with potent activity towards the PAC1 receptor. Peptides.

[78] Lambert GA, Truong L, Zagami AS (2011). Effect of cortical spreading depression on basal and evoked traffic in the trigeminovascular sensory system. Cephalalgia.

[79] Friedman DI, Quiros PA, Subramanian PS (2017). Headache in idiopathic intracranial hypertension: findings from the idiopathic intracranial hypertension treatment trial. Headache.

[80] De Simone R, Marano E, Bilo L (2006). Idiopathic intracranial hypertension without headache. Cephalalgia.

[81] Marucco E, Lisicki M, Magis D (2019). Electrophysiological characteristics of the migraine brain: current knowledge and perspectives. Curr Med Chem.

[82] Mínguez-Olaondo A, Quintas S, Morollón Sánchez-Mateos N (2022). Cutaneous allodynia in migraine: a narrative review. Front Neurol.

